# Confocal laser imaging in neurosurgery: A comprehensive review of sodium fluorescein-based CONVIVO preclinical and clinical applications

**DOI:** 10.3389/fonc.2022.998384

**Published:** 2022-10-03

**Authors:** Francesco Restelli, Andrea Maria Mathis, Julius Höhne, Elio Mazzapicchi, Francesco Acerbi, Bianca Pollo, Karl Quint

**Affiliations:** ^1^ Department of Neurosurgery, Fondazione Istituto di Ricerca e Cura a Carattere Scientifico (IRCCS) Istituto Neurologico Carlo Besta, Milan, Italy; ^2^ Department of Neurosurgery, Inselspital, Bern University Hospital, University of Bern, Bern, Switzerland; ^3^ Department of Neurosurgery, Universitätsklinikum, Regensburg, Germany; ^4^ Department of Neuropathology, Fondazione Istituto di Ricerca e Cura a Carattere Scientifico (IRCCS) Istituto Neurologico Carlo Besta, Milan, Italy; ^5^ Quint Healthcare, Fürth, Germany

**Keywords:** confocal laser endomicroscopy (CLE), confocal imaging, confocal laser microscopy (CLM), *in vivo* imaging, neurosurgery, brain tumors, sodium fluorescein

## Abstract

Given the established direct correlation that exists among extent of resection and postoperative survival in brain tumors, obtaining complete resections is of primary importance. Apart from the various technological advancements that have been introduced in current clinical practice, histopathological study still remains the gold-standard for definitive diagnosis. Frozen section analysis still represents the most rapid and used intraoperative histopathological method that allows for an intraoperative differential diagnosis. Nevertheless, such technique owes some intrinsic limitations that limit its overall potential in obtaining real-time diagnosis during surgery. In this context, confocal laser technology has been suggested as a promising method to have near real-time intraoperative histological images in neurosurgery, thanks to the results of various studies performed in other non-neurosurgical fields. Still far to be routinely implemented in current neurosurgical practice, pertinent literature is growing quickly, and various reports have recently demonstrated the utility of this technology in both preclinical and clinical settings in identifying brain tumors, microvasculature, and tumor margins, when coupled to the intravenous administration of sodium fluorescein. Specifically in neurosurgery, among different available devices, the ZEISS CONVIVO system probably boasts the most recent and largest number of experimental studies assessing its usefulness, which has been confirmed for identifying brain tumors, offering a diagnosis and distinguishing between healthy and pathologic tissue, and studying brain vessels. The main objective of this systematic review is to present a state-of-the-art summary on sodium fluorescein-based preclinical and clinical applications of the ZEISS CONVIVO in neurosurgery.

## Introduction

Despite recent therapeutic advances, the prognosis of brain tumors remains poor (1, 2). Surgical resection has a leading role in the treatment of brain tumors, given the results of different clinical trials that have shown that extent of resection (EOR) correlates with better outcomes, especially when combined with adjuvant therapies such as radiotherapy and chemotherapy. Nevertheless, it is well known that Gross Total Removal is not always possible and this aspect is mainly related to the fact that distinction between normal and pathologic tissue is often difficult, especially at the tumor margins (2–5).

Among the several tools and devices that have been implemented in recent years with the objective of increasing EOR, such as intraoperative ultrasound, neuronavigation, and the use of fluorophores, which can improve visualization of tumor tissue during surgery, showing to improve tumor margin identification and lead to more extensive resections (6–9), to date only histopathologic techniques can microscopically identify tumor cells and the actual infiltration at the tumor margins.

Histopathologic analysis remains the gold standard for definitive diagnosis, with frozen section role as the most rapid, used and diffused intraoperative histopathologic method that can offer intraoperative differential diagnosis. Nevertheless, the results obtained with frozen section analysis are often nondiagnostic or, worse, misleading, especially in cases of mechanical tissue destruction by the resection process (10–12). In addition, this method has other significant disadvantages. For instance, tissue sample analysis requires a long time and is usually performed outside of the operating room (OR). Moreover, the accuracy of this technology in determining diagnosis is also questioned due to a well-known diagnostic discrepancy between frozen sections and permanent sections of up to 2.7%, looking at intracranial pathologies (10). Such aspect is further complicated by the inherent heterogeneity of brain tumors. For instance, such tumors (i.e. gliomas) may contain high-grade populations embedded in a low-grade cell population and this aspect would be a significant challenge for the pathologist. For these reasons frozen section analysis still remains an unsatisfactory technology for revealing the histologic features necessary for the final diagnosis, especially if a task of “guiding intraoperative decisions” about EOR is wondered.

**Figure 1:**
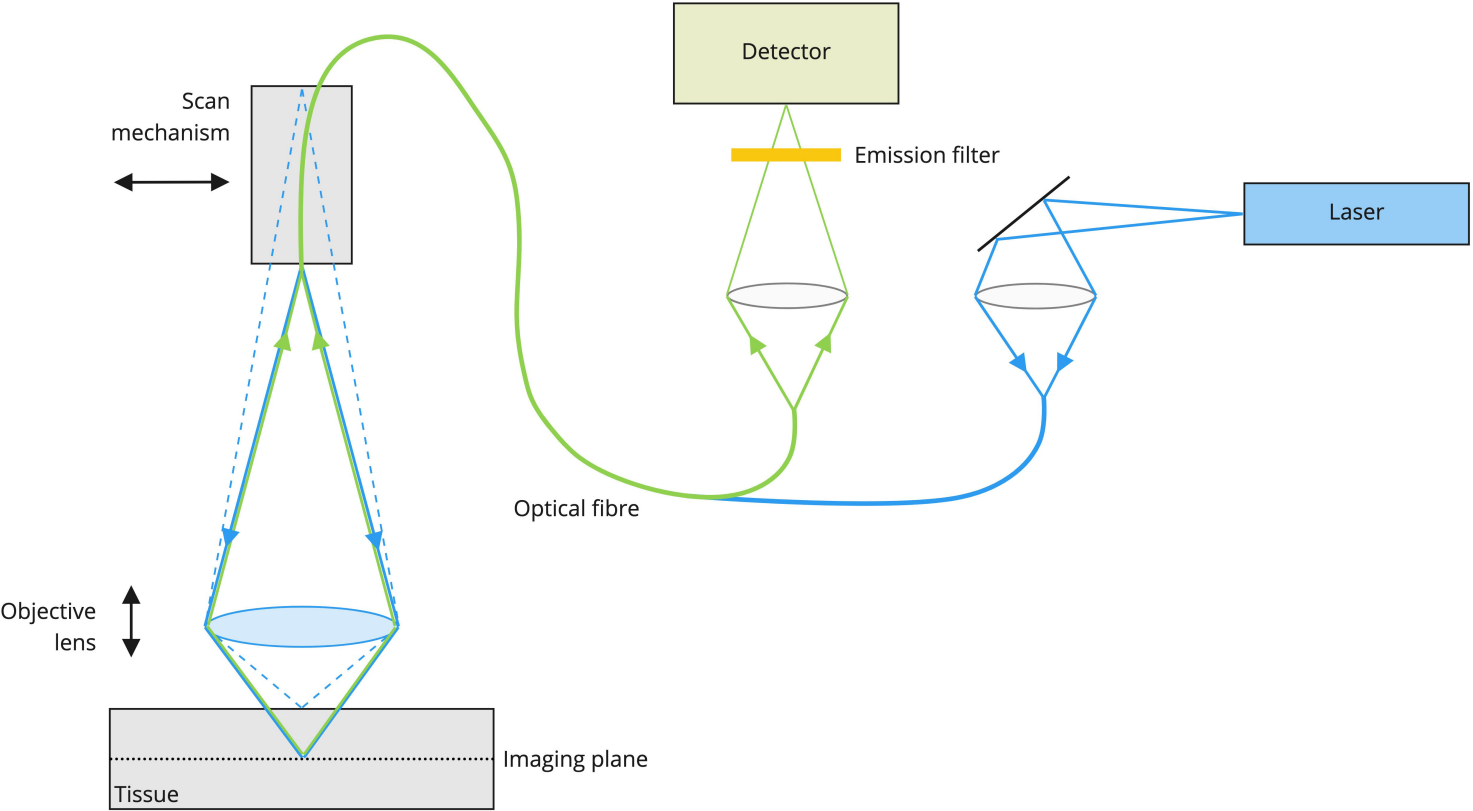
CONVIVO system mechanism of action. A laser-beam with a specific wavelength is focused on a point inside the object at a specific Z depth. A fluorescent dye that is in the tissue of interest is excited by the laser light and the fluorescence is collected by the lens system and focused onto the tip of the scanned delivery optical fiber, that acts as a confocal pinhole rejecting light other than that from the set Z depth. Then, the fluorescent light is carried to the confocal processor *via* the optical fiber through a fiber based optical coupler and into a detector, which synchronously samples the fluorescence providing an electrical representation of the light intensity that is recorded as a digital sample. The digital samples are constructed into an image frame that is sent *via* a digital interface to the integration computer. The integration computer uses custom host software to deliver the image data to a monitor for display and further analysis.

In this context, confocal laser technology demonstrated to be a technique that is able to provide real-time microscopic information about tissues and for such reasons it has already been included into common clinical practice in non-neurosurgical fields. Considering the technology, briefly, a laser source is used to deliver light *via* an optical fiber coupler and scanned delivery fiber to a lens system. The lens system that is mounted at the front of the scanner focuses the laser light into the sample to a depth set by a Z depth focusing mechanism integral to the scanner. A fluorescent dye that is in the tissue of interest is excited by the laser light. The fluorescence is collected by the lens system and focused onto the tip of the scanned delivery optical fiber. The optical fiber acts as a confocal pinhole rejecting light other than that from the set Z depth. The fluorescent light is carried to the confocal processor *via* the optical fiber through a fiber based optical coupler and into a detector. The detector synchronously samples the fluorescence providing an electrical representation of the light intensity that is recorded as a digital sample. The digital samples are constructed into an image frame that is sent *via* a digital interface to the integration computer. The integration computer uses custom host software to deliver the image data to a monitor for display and further analysis (**Figure 1**).

Confocal laser endomicroscopy (CLE) has been implemented with good results in general surgery, or in gastroenterology, urology, and gynecology, where very often a careful examination of pathologic margins is mandatory (13–16). In neurosurgery, CLE is still far from being routinely used, but in recent years it has been proposed in this field. The first studies in mouse glioblastoma (GBM) models were focused on distinguishing normal brain from microvasculature and tumor margins (17–19). After such initial preclinical experiences, the feasibility of CLE in human brain tumors was investigated both in *ex vivo* and *in vivo* studies with promising results (20–23). Second generation CLE systems, such as the ZEISS CONVIVO (Carl Zeiss Meditec AG, Oberkochen, Germany), have been specifically ideated for neurosurgical use and have undergone a deep investigation in recent years. CONVIVO was studied in animal models and in *ex vivo* and *in vivo* experiences, preliminary confirming its ability, when coupled to sodium fluorescein (SF) intravenous injection, in intraoperatively providing a large number of optical biopsies with imaging of cells at the microscopic/histologic level, representing the first technique able to provide real-time *in vivo* histopathological data from fresh tissue (24–29). Such aspects also lead to FDA approval of the machine for intracranial neurosurgical procedures in the US (30).

Overall, the neurosurgical literature suggests that this technology is capable of intraoperatively providing information regarding tumor tissue, both for diagnosis and for identifying tumor at periphery. Nevertheless, also due to the paucity of data available, the precise sensitivity, specificity, and accuracy in identifying tumor cells and the actual role this technology could play in neurosurgery soon are still under in-depth investigation.

The main objective of this systematic review is to present an update on the actual SF-based preclinical and clinical applications of the ZEISS CONVIVO in neurosurgery.

## Material and methods

### Literature search and screening process

A comprehensive literature search was performed in March 2022 and updated in July 2022 to include papers published since. MEDLINE (PubMed), EMBASE and SCOPUS were searched using the following search strings in the “Title/abstract” field: “confocal AND neurosurgery”, “confocal AND glioma”, “confocal AND brain tumor”, “endomicroscopy AND neurosurgery”, “endomicroscopy AND brain tumor”, “endomicroscopy AND glioma”, “confocal imaging AND glioma”, “confocal imaging AND brain tumor”, “confocal imaging AND neurosurgery”, “confocal endomicroscopy AND glioma”, “confocal endomicroscopy time limits AND brain tumor”, “confocal endomicroscopy AND neurosurgery”, “Convivo AND glioma”, “Convivo AND brain tumor”, “Convivo AND neurosurgery” (published article until July 15th, 2022).

Search was limited to articles in English. All titles and abstracts were checked by two different researchers (F.R. and K.Q.). Frank duplicates were removed. Relevant works were collected, organized, and studied. Furthermore, bibliographies were hand-searched to identify further relevant literature.

If there was a difference in opinion on appropriateness of the works among the researchers, a consensus was reached consulting a third reviewer (A.M.). In order to further broaden the search process for studies that might have been missed through the first search, during this first-phase pure reviews on the topic were not excluded a priori. Given the large differences in patients’ cohorts and methodologies used in the different studies analyzed, the literature search did not strictly follow the criteria for a systematic review, therefore trying to identify the highest quality of available evidence for each specific theme.

### Eligibility criteria

After the screening process, remaining articles were completely read and analyzed by two authors (F.R. and K.Q.). The authors checked for their relevance and eventual accordance with our inclusion and exclusion criteria. In particular, only studies concerning *in vivo* or *ex vivo* applications of CONVIVO confocal imaging technology coupled with intravenous SF administration in neurosurgery were analyzed. We decided to include in the review also the clinical results of preclinical works (works with both a preclinical and a clinical experimental part). The following inclusion/exclusion criteria were applied:

Inclusion criteria:

-Clinical works based on SF-CONVIVO imaging technology applications in neurosurgery;
-preclinical works with some clinical results related to SF-based CONVIVO confocal imaging technology in neurosurgery;
-case reports, in which SF-based CONVIVO imaging was performed.

Exclusion criteria: Correspondences, Comments, Letters to the Editor, Proceedings and Conference Papers, purely preclinical studies.

### Data extraction

All included studies were extracted and summarized in tables. Authors, year of publication, journal of publication, type of study, CLE system used, fluorophore used, dosage and timing, fluorophore re-administration, number of cases, tumor type(s), study description, main findings and results related to diagnostic performance from each study were reported. Due to the large heterogeneity of the available and identified studies, considering also the limited number of published works, we present the data as a narrative review.

### Statistical analysis

Statistical analysis of the data, for the purpose of a meta-analysis, was not possible due to substantial heterogeneity in study design and populations.

## Results

**Figure 2:**
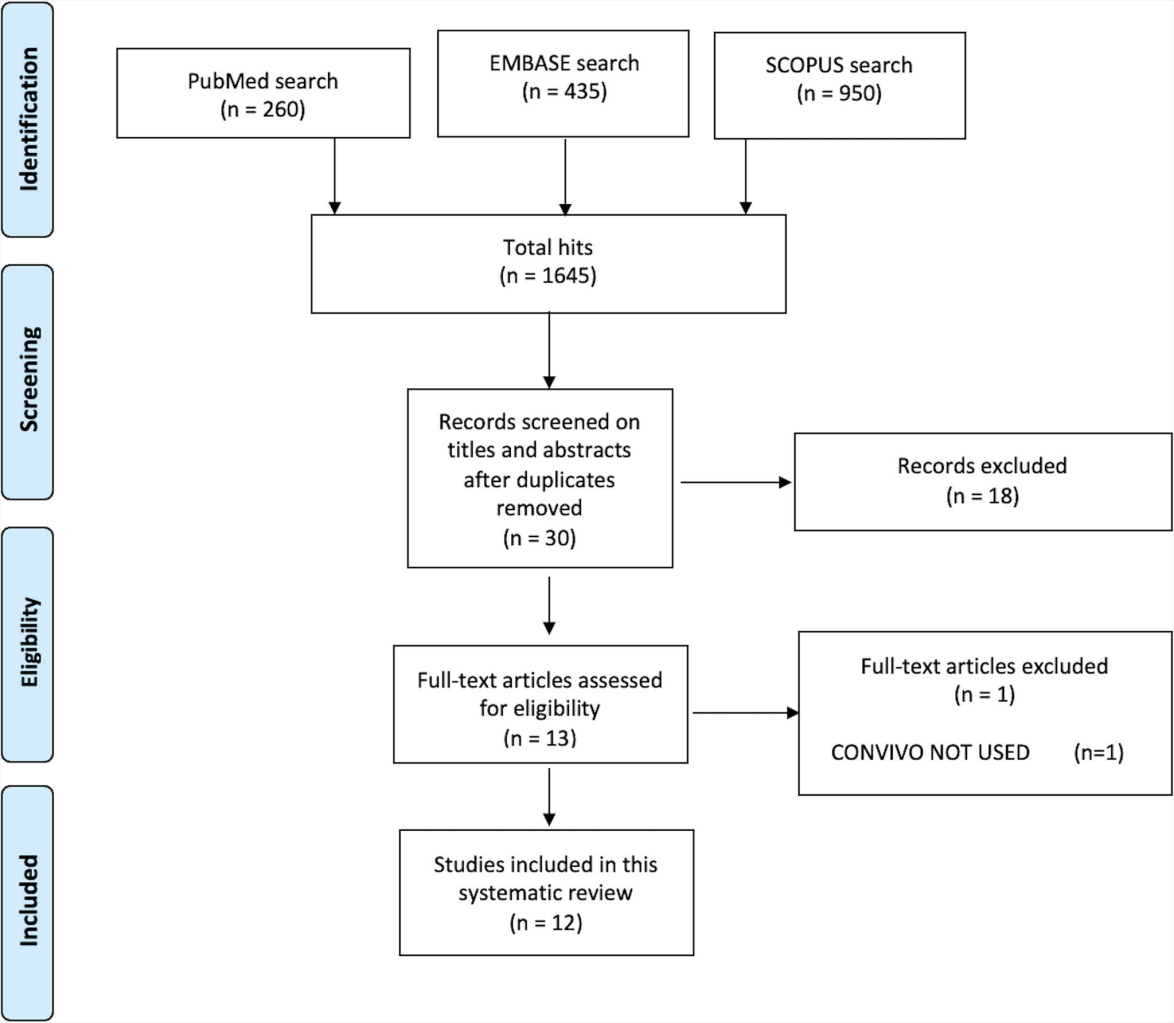
The flowchart of search hits and the different Preferred Reporting Items for Systematic reviews and Meta-analyses (PRISMA)-guideline selection phases, from the initial search and the follow-up search (B), resulting in the total 12 included articles.

A total of 1645 hits were found by the first search among the three Databases (Pubmed 260, EMBASE 435, SCOPUS 950). Among the works we found, 30 works were completely screened reading titles and abstracts, removing duplicates. Finally, 12 full-text articles were considered for eligibility, finding all of them suitable for the final review analysis (**Figure 2**).

### Preclinical studies

The CONVIVO system, designed specifically for neurosurgical use cases, was developed based on a first-generation CLE system designed for gastrointestinal use (Optiscan Pty., Ltd., Mulgrave, Australia). In a rodent glioma model study, Belykh et al. investigated performance improvements of the CONVIVO system (Gen2) compared to the Optiscan system (Gen1) (25). Performance in visualization of vessels, normal brain and tumor cells was similar with both systems. Compared to Gen1, Gen2 showed a smaller field of view, but much higher image resolution and better image quality. Further advantages of the Gen2 compared to Gen1 were a more friendly user interface, metadata handling and image transfer process. Gen2 moreover offers a z-stack imaging mode, enabling 3D visualization of tissue areas. In the scope of this study, they administered different concentrations of SF and showed that overall performance is improved when using higher dosages (20 and 40 mg/kg vs. 0.1–8 mg/kg).

In a work from 2018, Belykh et al. investigated the diagnostic accuracy of *in-vivo* CLE in identifying different types of brain tissue (normal brain, injured brain and brain with tumor tissue in a mouse glioma model) (26). Ten female, 10-week old mice were injected with mouse glioma cells to establish a glioma model according to a previously defined protocol (17). Animals were injected with 1 mg/mL (n=3), 0.1 mg/mL (n=4) or no SF (n=3). Using the CONVIVO system (Carl Zeiss Meditec AG) imaging was performed 15 to 60 minutes after SF administration and 10 to 30 minutes after injury to normal brain, at known locations of 1) tumor (n = 60) and 2) injured normal brain (n = 25), in animals administered with SF; and 3) normal brain tissue (n = 5) in control animals (no SF administration). A set of CLE images (n = 40) was given to trained experts for assessing type of tissue (1, 2 or 3). As reference served the diagnosis based on H&E image of correlative specimens. Mean accuracy for correctly differentiating tumor from injured or non-tumor tissue was 85%. Accuracy, specificity, and sensitivity for discriminating tumor from non-tumor tissue was 90%, 86% and 96% respectively.

### *Ex vivo* experiences

In 2018 Belykh and colleagues performed an interesting work where they obtained CLE imaging, Z-stack acquisition, and 3D image rendering of 31 human tumors. In this analysis meningiomas, gliomas, and pituitary adenomas were analyzed *ex vivo*. In this work, for the first time, the CONVIVO system (Carl Zeiss Meditec AG), was used to image human tissue (31). In this specific work, 2-5 mg/kg of SF were administered intravenously 5-60 minutes before imaging in 22 out of the 31 patients in total. Biopsy specimens obtained in the fluorescent tumor areas of patients who received SF intraoperatively were then imaged in the operative room with the help of a stand-alone CLE system within 1–10 minutes after specimen acquisition. No further data on SF protocol of administration were given. Comprehensively, Belykh provided detailed 3D images of different kinds of brain tumors, suggesting that this technology might allow for an increased spatial understanding of tumor cellular architecture, also increasing visualization of surrounding related structures compared with two-dimensional images.

Some years later, the same group used CONVIVO on 47 patients with a total of 122 biopsies analyzed (29 HGGs) (28). The authors were interested in performing a sensibility/specificity study, using a classical SF administration protocol (SF 2 mg/kg for patients with gliomas and meningiomas, SF 5 mg/kg in patients with metastasis). Comprehensively the authors found a positive predictive value of CLE optical biopsies of 97% for all specimens, while a positive predictive value of CLE optical biopsies of 98% for gliomas. Specificity was found to be 90% for all specimens and 94% for gliomas. Furthermore, the authors described improved image quality percentage of accurately diagnosed images (67% vs. 93%) in those cases where a second SF injection was performed during the surgery (after a mean of 2.6 h after the first injection, 5 mg/kg intravenously upon request), suggesting for the first time that a re-administration of SF during the surgical procedure may increase the diagnostic value of the images taken with CONVIVO.

In 2019, the group of Schebesch reviewed their recent experience in a neuro-oncology center, demonstrating the possibility of operating while combining different imaging modalities intraoperatively. They presented three cases with an *ex vivo* analysis by CONVIVO with a 5 mg/kg SF protocol at anesthesia induction (a supratentorial astrocytoma WHO III, a motor area glioblastoma WHO IV and an oligodendroglioma WHO grade III). All these cases were managed combining different visualization modalities, such as high-definition endoscopes, fluorescence-guided surgery and confocal endomicroscopy with CONVIVO. Besides indicating the dosage used, no further details of the imaging procedure were reported (32).

In 2020, the group of Acerbi and colleagues studied the ability of Convivo in offering an intraoperative first-diagnosis during GBM removal *ex vivo*. The authors blindly compared intraoperative CLE and frozen/permanent sections results at both central core and tumor margins of tumors (29). In this specific context, the main objective of the authors was to both check for CONVIVO ability in offering an intraoperative diagnosis and in categorizing morphological patterns (i.e. cellularity, vascularization and necrosis). SF was administered following Acerbi and colleagues recommendations regarding SF usage in neuro-oncological surgery (29). Five mg/kg of SF at anesthesia induction permitted an acceptable identification of tumor tissue during the resections, allowing also to perform CONVIVO analysis. In fact, blindly comparing CONVIVO and frozen sections images a high rate of concordance in both providing a correct diagnosis and categorizing patterns at tumor central core (80 and 93.3%, respectively) and at tumor margins (80% for both objectives) was disclosed. Lower rates of concordance were found if compared to permanent sections (total/partial concordance in 80 and 86.7% for diagnosis and morphological categorization, respectively).

In 2021, Abramov and colleagues investigated the effects of redosing SF on CLE image quality and diagnostic accuracy. They retrospectively analyzed *ex-vivo*-obtained CLE images from patients resected with SF-based fluorescence guidance (33). SF was administered at anesthesia induction (2 or 5 mg/kg with possibility of redosing in case CLE images brightness was considered inadequate by the neurosurgeon). Three groups of CLE images were analyzed: CLE images acquired from patients after initial dosing (initial-dose group, n = 6), after redosing once (redose group, n = 6), and images from patients without a redosing (single-dose group, n = 9). Images were compared for brightness and contrast, image quality, and qualitative image assessment and diagnostic accuracy by 7 reviewers with different levels of experience. Brightness and contrast of the images were not significantly different when SF was administered at 2 or 5 mg/kg. Across the image groups, brightness and contrast were significantly higher in the redose group vs. initial-dose group and in the initial-dose group vs. the single-dose group (p < 0.001 for each). In matched analysis between the initial-dose imaging group vs. the single-dose imaging group, this could be attributed to the timing of the imaging (93.9 ± 50.1 minutes vs. 123.2 ± 35.9 min, p = 0.002). A moderate correlation between the timing of imaging and image brightness and contrast of the CLE biopsies was also found (brightness: rho = -0.52, p < 0.001; contrast: rho = -0.57, p < 0.001), indicating that image acquisition early after SF administration leads to a better image quality. Qualitative image assessment revealed the highest scores in the redose group, followed by the initial-dose and the single-dose groups. Diagnostic accuracy in the redose group, in which images were acquired at a mean of only 6.4 minutes after SF redosing, was 83% regardless of reviewer experience. The time-dependent kinetics and limited signal duration of SF fluorescence resulted in darker images and worse contrast with increasing imaging time, which ranged between 3 and 180 minutes in this *ex-vivo* study.

Belykh and colleagues from the group of Mark Preul undertook a feasibility study for CLE imaging of pituitary adenomas in 2020 (34). In a first feasibility approach, the CONVIVO imaging probe was successfully introduced through the transnasal transsphenoidal corridor in cadaveric specimens and was deemed adequate for imaging of the pituitary area. Secondly, resected human pituitary adenoma tissue samples were imaged *ex-vivo* and compared against standard H&E histology and/or frozen sections. CLE images resembled the tissue and cellular features known from standard histology, showing cells with prominent nuclei, non-organized tissue structure, vascularity, and stroma. There was a heterogeneous uptake of SF that created a nuclear/cytoplasmic contrast along with a contrast between neighboring cells. Depending on the classification used (tissue description or definitive tumor diagnosis), the concordance of the CLE biopsies with either frozen section or permanent histology ranged between 53.8% and 100%. Details of the analysis are described in **Supplementary Table 1**. Some CLE images were classified as non-diagnostic due to very early (< 1 minute) or late (> 10 minutes) acquisition following SF administration, leading to suboptimal contrasting of the cellular outlines. Other reasons for nondiagnostic images included erythrocyte contamination obstructing the field of view or too small physical samples, which prevented finding an optimal imaging spot.

An interesting case report was published by Belykh and colleagues in 2021 of a patient with an non-enhancing WHO II/III anaplastic oligodendroglioma, predominantly low-grade with high-grade foci of hypercellularity and increased mitotic figures (36). The patient received a single dose of 40 mg/kg of SF at the induction of anesthesia and was subsequently resected using fluorescence-guided surgery using a Yellow 560 filter. CLE images were recorded *ex-vivo*. This dose produced a bright signal and excellent CLE images of extremely clear cellular architecture with mitotic figures, endothelium and axons. A distinct morphologic appearance, not commonly observed with lower-dose SF were observed with the brightness and clarity of the CLE images, especially at the prolonged imaging time of up to 1.5 hours. Besides the typical yellowish skin discoloration, which resolved quickly, no side effects were reported. Besides the higher than usual administered dose of SF, the authors found abnormalities in the preoperative T2/FLAIR signal surrounding the tumor mass, which may be sensitive markers of a damaged blood brain barrier, contributing to an extravasation of SF in this predominantly low-grade oligodendroglioma. In the end, the authors discuss the utility of having a higher dose of SF in those cases where only one dose is planned to be administered at the beginning of an operation and they suggest it as an appropriate approach in those cases where using sensitive imaging such as CLE for discriminating the histoarchitecture of tumor margins may be of help, for instance for LGG tissue that may not be as amenable to 5-ALA fluorescence guidance.

### *In vivo* experiences

To date, three *in vivo* studies have already been performed.

In 2021, Höhne and colleagues published a study on feasibility, safety and potential applications of CLE (35). They performed SF-FGS and CLE-imaging in 12 patients with various CNS malignancies by using 10% SF at a dose of 5 mg/kg. The time between SF-administration and CLE-imaging varied between 10 - 120 minutes. Digital biopsies were taken at the tumor border, tumor center and the perilesional zone, defined as the infiltration/edema zone where the fluorescence signal started to become faint. The digital biopsies were compared against standard H&E histology. The authors reported a seamless integration of CLE-imaging in the surgical flow. As the CLE-probe is similar to other commonly used microsurgical instruments, CLE-imaging could be performed safely without traumatizing healthy tissue. Macroscopic SF-fluorescence was observed and considered helpful guidance in all cases. In CLE-imaging, all tumors (12/12) stained positively for SF at the tumor border, 11/12 at the tumor center and 7/12 in the perilesional zone. At the weight-adapted dose of 5 mg/kg, a shorter time between SF administration and CLE-imaging resulted in more assessable images. No major side-effects related to the use of SF were observed. The authors concluded CLE to be safe and feasible, and that further prospective trials are needed to confirm its promising potential.

Belykh and colleagues investigated the feasibility of CLE to qualitatively and quantitatively analyze real-time blood flow patterns in brain under normal conditions, after injury and in pathologic brain and spinal cord microvasculature in a large animal model and patient samples (27). In the swine model, SF concentrations ranged from 1 - 5 mg/kg and 0.1% - 0.005%/5 ml. In human patients, 5 ml of 10% SF was administered 5 minutes prior to CLE imaging and a total of >20,000 digital CLE biopsies obtained *in-vivo* or *ex-vivo* were analyzed. Around 8 minutes after SF administration in the animal model, arterial and venous capillaries and vessels between 5 - 250 µm in diameter could be visualized. CLE visualization time extended up to 30 minutes after initial administration and for up to 3 hours when reinjecting SF. They observed a SF-based contrast in the intravascular compartment, in the vessel wall and also in the perivascular parenchyma, when the blood brain barrier was disrupted. This allowed appreciation of vessel wall cellularity, the distinction between arterial and venous vasculature and the vasculature’s functional status. They observed that the fluorescence lasted longer than the intravascular contrast visible through the wide-field operation microscope. Both tissue injury, contrast extravasation, and additional injections of SF rendered visualization of the wall of the vessels much easier, rendering the vessel wall clearer at later imaging times. Intravascular events, such as the dynamics of thrombus formation during circulatory arrest, could also be observed. Additionally, lymphatic vessels in the dura could be visualized. In human samples of grade 2 and 3 astrocytomas and oligodendrogliomas and grade 4 glioblastomas, CLE visualized both normal and abnormal microvasculature. Abnormal microvasculature was characterized by disorganized nonlinear appearance and perivascular crowding of cells. Also slow or stagnant flow, perivascular leakage of fluorescent contrast, and cells attached to the inner vascular wall were observed. All such features were clearly visible in CLE images. For clinical considerations, CLE with SF allowed a substantially longer observation of blood flow compared to wide-field ICG or SF. The authors suggested potential use cases for CLE-based SF visualization of vasculature for traumatic brain injury and cerebrovascular lesions (for instance also analyzing the downstream effects of surgical vessel anastomoses or reconstruction), for flow recovery study after stroke, to study perforating vessel competency in vascular cases, studying flow dynamics in moyamoya disease/syndrome, and revealing tumor blood vessel and flow characteristics in oncological cases.

The most recent study by Abramov and colleagues from 2022 aimed to evaluate the *in vivo* safety and feasibility of the ZEISS CONVIVO for intraoperative application in human brain tumor surgery (24). The prospective 30-patient study used 5 mg/kg i.v. of SF given upon the surgeon’s request within five minutes prior to imaging. Due to the sufficient quality of the resulting images, no redosing was performed. Entities included 13 gliomas (WHO I, III, and IV), 5 meningiomas (WHO I and II), 6 other primary tumors (all WHO I), 3 metastases (breast, kidney, and lung tumors), and 4 cases with reactive brain tissue following previous resection, chemo- or radiotherapy. CLE images were assessed against frozen sections and permanent histology. Across all samples, the diagnostic accuracy, sensitivity, and specificity reported for CLE vs. frozen section was 94%, 94%, and 100%; for CLE vs. permanent histology 92%, 90%, and 94%, respectively. A neuropathologist could interpret the CLE images in 97% of cases (29/30). Interpretable images were obtained within a mean of 6 images and within the first 5 seconds of imaging. Interpretable image acquisition was positively correlated with study progression, number of cases per surgeon, cumulative length of CLE time, and CLE time per case.

## Discussion

**Figure 3:**
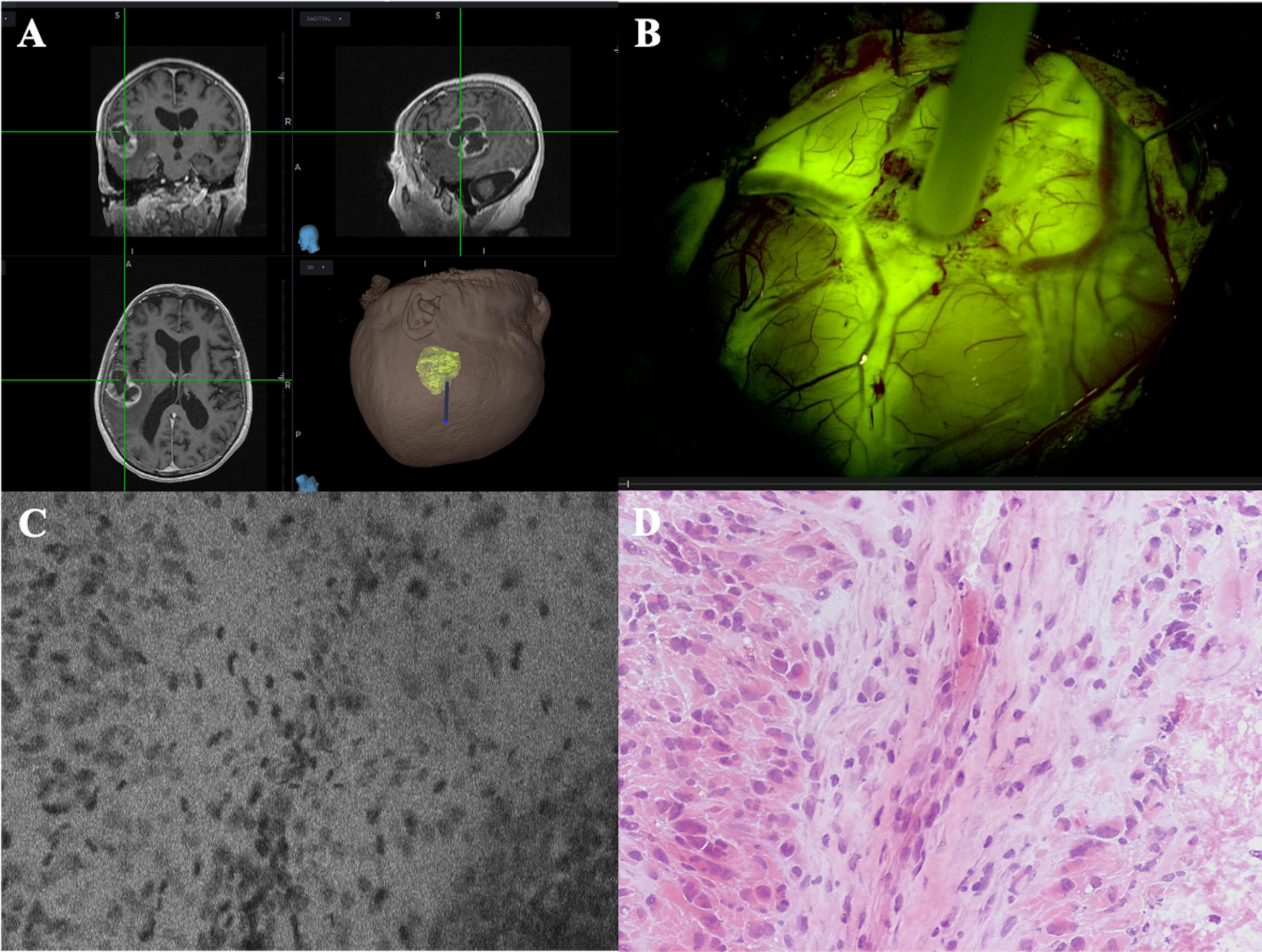
Case example of an *in vivo* GBM case analyzed with CONVIVO (courtesy of Dr. Acerbi and Dr. Pollo, Fondazione IRCCS Istituto Neurologico Carlo Besta, Milan, Italy). **(A)** MRI preoperative images of a left parieto-temporal GBM, loaded on Stealth S8 navigation system (Medtronic, Minneapolis, USA). **(B)** CONVIVO stylet placed upon the center of the tumor, on the cerebral surface. As it can be seen, the tumor intensely enhances after intravenous SF administration. **(C, D)** CONVIVO and histological images of the point where the optical biopsy with CONVIVO was obtained. Disordered groups of dark nuclei cells can be seen, along with a stromal component among them. A low fluorescence area on CONVIVO, as it occurs in necrotic parts of the tumor, can be seen in the bottom right of panel **(C)**, with its histological counterpart in the bottom right of panel **(D)**.

In this work we have collected and reviewed the available literature on preclinical and clinical protocols for the application of SF in confocal endomicroscopy (**Supplementary Table 1**). We have focused on the FDA-approved device ZEISS CONVIVO, which has been designed and dimensioned specifically for use in neurosurgical applications (30). It can record digital *in vivo* and *ex vivo* tissue biopsies in real-time, prior to tissue resection, thus adding an important new tool to the neurosurgeon’s and neuropathologist’s armamentarium (**Figure 3**).

### Previous studies using CLE in neurosurgery

Starting from the works published on prototype and technically compatible devices to the CONVIVO, such as the devices by OptiScan, may help in comprehending the great interest that such technology is keeping among the neurosurgical community. An initial *ex vivo* clinical study using 0.05% topical acriflavine was performed with a miniaturized confocal laser microscope from OptiScan (37). This study showed a high degree of concordance in histopathologic diagnostic criteria for glioblastoma, such as cell number and density, cell pleomorphism, mitotic figures and rate of mitosis, microvascular proliferation, and pseudopalisading necrosis. Depending on each criterion, the tumors showed various degrees of correspondence between confocal imaging and histopathology (cell density and pleomorphism in all 100% of tumors, microvascular proliferation in 44% and mitotic figures and necrosis in 22%). Cell density analyses allowed the authors to differentiate tumor center areas from the infiltration zone on confocal images alone. In just one case, confocal images were perfectly corresponding to histopathology in all five diagnostic aspects. Due to the mutagenic effects observed for acriflavine, subsequent studies were performed with SF due to its standard usage in ophthalmology and its advantageous safety profile.

Among these studies, Eschbacher et al. have used 25 mg/kg of SF administered i.v. at the time of tumor exposure, with *ex vivo* imaging being initiated within two to five minutes and lasting from two to ten minutes (38). The study design allowed direct comparison of CLE images with standard histology. The CLE biopsies correlated well with the traditional histological findings across a variety of tumor types. Pathognomonic cytoarchitectural features could be visualized by CLE as well. Overall, 92.9% (26/28) of lesions were correctly diagnosed by CLE alone in a blinded analysis, well within the range of diagnostic accuracy between 92% and 99.7% reported for frozen sections and standard histology (10–12, 39, 40). This seminal work of Eschbacher et al., in particular, describes in detail the morphological appearance of meningiomas, schwannomas, low- and high-grade gliomas, ependymomas and hemangioblastomas, and prepared the ground for tumor-specific criteria for CLE image interpretation.

### Clinical use and potential applications of new-generation CLE technology in neurosurgery

Second generation CLE systems, such as the ZEISS CONVIVO, have been specifically ideated for neurosurgical use and have undergone a deep investigation in recent years, preliminarily confirming their ability, when coupled to SF intravenous injection, in intraoperatively providing many optical biopsies with histological resolution, representing the first technique able to provide near real-time *in vivo* histopathological data from fresh tissue. Such a new-generation system has undergone a deep investigation in recent years, as anticipated above, due to multiple reasons. Intraoperatively, the time until a neuropathological diagnosis is received could be greatly shortened. The sensitivity and quickness in having an answer of such a system could influence neurosurgical decision making, particularly at the presumed margins of a tumor resection cavity. Real-time *in vivo* histology could contribute to a better and quicker visualization of the tumor border at the microscopic level, inspecting eloquent tissue for tumor invasion, and possibly augmenting current standard fluorescence-guided surgery practices. Mistakes and incorrectness related to sampling procedures are common issue during frozen-section analysis. These aspects could be lessened with real-time examination of specimens. *In vivo* confocal microscopy could also favor the selection of areas characterized by highly cellular tissue, facilitating histological diagnosis, molecular testing and eventual tissue banking for downstream diagnostic workflows. Also common confounding factors, such as frozen-section artifact and cautery artifacts may also be avoided when applied in lieu of frozen-section. Moreover, because all data are digitally acquired and stored, electronic transmission of images to remotely located neuropathologists could enable a real-time telepathology with benchside diagnosis. Lastly, the readily available digital images can be used for advanced image analysis using artificial intelligence, known in radiology as radiomics or radiogenomics. Exemplarily, using MRI imaging data and convolutional neural networks, a review of fourteen studies reported a sensitivity of 94% and specificity of 87% for classifying the IDH status, and 90% sensitivity and 89% specificity for assessing the 1p19q codeletion status in WHO grade II/III tumors. Whether a similar approach can be achieved using digital histologic imaging remains to be investigated. CLE technology could be a door opener to such advanced diagnostic approaches.

### Safety profile of sodium fluorescein

As anticipated, the technology that resides under the possibility of looking at a cellular level with the ZEISS CONVIVO is based on SF administration. Looking at SF, as anticipated, this dye recently gained great interest in the neurosurgical community for oncological and neurovascular applications. In particular, its ability to accumulate in cerebral areas where a damage to the BBB has occurred allows the dye to concentrate at tumor sites, rendering tumor tissue more visible, particularly if a dedicated filter on the surgical microscope is equipped (41). One of the main reasons for the widespread use of SF is, besides its proven ability to increase GTR rates and a very affordable low cost (around 5 Euros per vial), its well described safety profile, as confirmed by several years of application in general surgery, gastroenterology, and especially in ophthalmology (42). Looking at the safety profile, most reports of allergic reactions due to SF are related to angiographies for vitreo-retinal pathologies. These sporadic patients are generally affected by mild allergic reactions, like nausea and vomiting, sneezing and pruritus, rather than severe, life-threatening ones, like laryngeal edema, seizures or circulatory shock. This aspect was confirmed in the previous years by various works (42, 43). In neurosurgical literature, we couldn’t find structured reports of side effects other than isolated severe ARs reports (44–47). This aspect may be due to unidentified cases but also to unreported events. Nevertheless, almost every study where SF was used in neurosurgery, either for oncological or neurovascular cases, has always underlined the totally safe profile of this dye, even for high doses, also considering that in recent years the development of specific filters for surgical microscopes (Pentero with YELLOW560 filter, Carl Zeiss Meditec AG) allowed a reduction in SF doses necessary to enhance tumor tissue during oncological surgeries from 10-15 mg/kg to dosages around 5 mg/kg (48). As expected, such safety data have been confirmed by the articles we reviewed. No serious adverse events were encountered in the published CONVIVO series, apart from yellow-colored urine and, in some patients, yellow tinging of the skin that usually resolved in all series within 24 to 48 hours. This aspect remained true even when considering those works, in which SF was administered at 40 mg/kg (36), or in which SF was voluntarily re-administered (33). In fact, starting from this point, some authors suggested studies with higher SF doses due to this established safety profile.

### Sodium fluorescein clinical protocols in neurosurgery and in CLE imaging

Looking at the possible use that SF may have in neurosurgery, researchers have studied multiple uses of SF, in particular to demarcate tumor borders and to help in achieving gross total resection (41). Starting from the early experiences of Shinoda and colleagues in 2003, where high doses of SF (up to 20 mg/kg) were used, due to the lack of special filters equipped on surgical microscopes (49), the current trend consists in lower dosages (around 5 mg/kg), due to the progressive availability of microscopes equipped with special filters specific to the wavelengths required for SF. Various reports of SF use in vascular surgery include examination of flow dynamics in arteriovenous malformations before and after exclusion of arterial feeders, pre and postoperative study of intracranial aneurysms, cortical microcirculation imaging, assessing of anastomotic patency in revascularization procedures, and analyzing flow in perforating arteries in proximity to aneurysms. Such aspects are usually studied with different administration protocols, that range around the administration of a bolus type of injection, on demand, of around 500 mg of SF (50).

Different points regarding administration protocols should be raised when it comes to SF protocols in CLE. In fact, the difference should be underlined between a correct SF administration timing for a neuro-oncological purpose (i.e. to increase EOR) versus the best timing for obtaining clear CLE images. Regarding the first point, the group of Acerbi and colleagues already pointed out that time of injection is a fundamental aspect to allow an optimal discrimination between tumor and peri-tumoral areas. In particular, it was suggested to implement a low-dose (5 mg/kg) i.v. administration of SF at the end of patient intubation (i.e. around 1 h before dural opening). In fact, with this timing of injection a good discrimination of fluorescent and non-fluorescent tissue may be obtained, with consequent high rate of GTR for HGGs (41). In fact, one of the issues of injecting SF in an acute way (for instance, on demand during neurovascular surgeries) is that this methodology leads to an intense fluorescence uptake, even by normal brain tissue, because of the passage of SF throughout small capillary vessels (50). This type of bolus injection has therefore been advocated only for vascular indications, as for aneurysms or arteriovenous malformation surgery (see above), similarly to what is carried out, normally, for indocyanine green injection, with good results (51).

**Figure 4:**
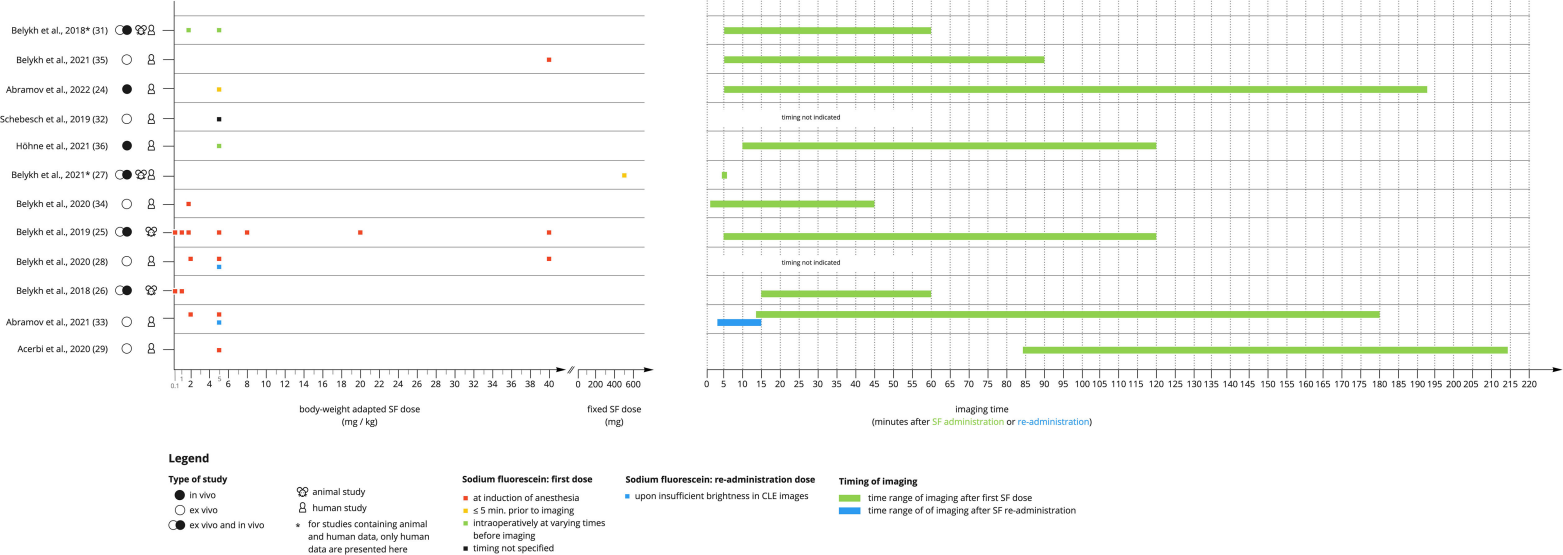
Graphical visualization of SF dosing protocols and timing of imaging in the different studies analyzed.

Looking at SF protocols of administration in CONVIVO imaging, it must be said that most of the authors are keeping a somewhat similar protocol of administration, with SF being given at patient intubation, following neuro-oncological purposes (**Figure 4**). Acerbi and colleagues studied 15 GBM cases in 2020 using the well-established 5 mg/kg protocol at anesthesia induction, and no re-administration. In this specific case, time from SF injection to CONVIVO scanning was higher, up to a mean of 137.96 min. for biopsies taken at the tumor core and 130.76 min. for biopsies taken at tumor margin with a mean value of 134 ± 31 minutes (122–214 min), taken together (29). In Höhne and colleagues’ *in vivo* experience, a weight-adapted dose of 5 mg/kg of SF was administered intravenously prior to imaging and the timing varied between patients. It was observed that a shorter elapsed time correlated to more readable and assessable images (35). In both *ex vivo* works of Belykh from 2018 and 2020, a 2-5 mg/kg of SF administration around one hour before imaging was executed with good results in terms of tumor visualization (26, 28). In particular, in the 2020 work, the authors noticed that, in many cases, biopsy acquisition occurred more than 90 minutes after the first SF administration, which resulted in suboptimal contrast in CLE images, and such decrease in image quality was also found for biopsies when the SF was injected 1 to 5 minutes before imaging (28). Nevertheless, when considering the analysis of all biopsies, as well as the glioma-only biopsies obtained at different time points after SF injection, no correlation between the timing of SF administration and image quality was shown (20). Interestingly, when 40 mg/kg were administered to a patient, CLE demonstrated high-quality images with excellent contrast in visualizing tumor cells, supporting the idea of higher doses or re-administration during surgery. A confirmation of this aspect was then given in two subsequent and recent works from Abramov and Belykh in 2021 (33, 36). In the first work, the administration of 40 mg/kg of SF at anesthesia induction in a low-grade glioma patient improved CLE visualization of tumor cellularity, while in the second work a retrospective comparison was performed between *ex vivo* images acquired after SF redosing, images from the same patients acquired after the initial SF dose (initial-dose imaging group), and images from patients in whom redosing was not used. Interestingly, the authors found that the brightest and most contrasting images were taken in the redosing group if compared to the initial-dose and single-dose groups (p < 0.001). The decay of SF signal resulted to be negatively correlated with brightness and contrast. It was also found that as the mean timing of imaging increased, the percentage of accurately diagnosed images decreased (p = 0.03).

Considering all the works together, apart from their *ex vivo* or *in vivo* nature, and apart from the time from SF to surgical resection that could be necessary following the “neuro-oncological purpose” specified above, it seems that clearer CLE images can be obtained when shorter times between SF administration and CLE imaging and higher doses of SF are taken into consideration, but the exact timing seems to be dependent on the specific tumor type. Nevertheless, at the present time, we feel it is too early to state if there is a “best option” for each case. As mentioned before, giving higher SF dosages may increase readability of CLE images, but, in turn, rendering tumor removal more difficult due to the lack of tumor specificity of SF. On the contrary, keeping lower SF dosage protocols may improve this aspect at the expense of CLE image quality. Further research is needed to highlight pros and cons of the different approaches, trying to find an algorithm that may help surgeons in choosing the correct dosage for each specific case.

Looking at the only available CONVIVO vascular experience by Belykh and colleagues from 2021, patients received 5 ml of 10% SF i.v. 5 minutes prior to CLE imaging (27). The earliest visualization of the vessel wall was 8 minutes after SF injection, with visualization being more common at approximately 30 minutes after injection. As expected, tissue injury, contrast extravasation, and higher dosages (i.e., additional injections of SF) resulted to be directly correlated with easier vessel wall visualization. After intravenous injection of SF, fluorescence intensity was strong enough for CLE intravascular imaging for at least 20-30 minutes and adequate for longer imaging after subsequent SF injections. Lastly, blood flow could be visualized continuously within a total time of more than three hours of imaging when reinjecting SF (27).

As a further step, we performed a brief online survey among the clinical users of the CONVIVO around the world, regarding their experiences in appropriate dosage and timing of SF for CLE, of which we report just a narrative recap.

We found each center currently using SF dosages that strictly follow local institutional guidelines for vascular or neuro-oncological use (max. 500 mg). High regulatory burdens hamper the evaluation of higher dosages in clinical trials. The timing of the i.v. injection however varies among centers. For instance, most centers usually inject a single dose at the time of skin incision or dural opening and a few others administer a single dose 5 - 15 minutes before the intended imaging time. These differences result in time delays from injection to imaging of about 5 to 60 minutes. All investigators reported good quality images with their protocols, which suggests that timing does not completely correlate with image quality at this specific point, reflecting the findings of some authors (31, 51), but at the same time raising questions on the possibility and necessity of creating a “standard” injection protocol with standard doses and timing of injections. Probably, most of the reasons for these questions find an answer in the necessity of following a clear clinical question (such as: need to identify the tumor border?; need to make a diagnosis?; need for increased contrast in a lower grade tumor)?. As a matter of fact, one of the hot topics that still needs to be better studied and defined is the appropriate SF injection protocol, especially considering its timing when looking for a tumor margin. While for fluorescence-guided resection early administration of SF is recommended to achieve proper demarcation of tumor versus non-tumor tissue by the degree of SF extravasation, as stated above, late SF administration with CLE enables demarcation based on the cytoarchitectural structure, which seems to be prioritized among the community of pathologists. Whether this is widely applicable in practice remains to be clarified and results shall be regarded when defining the SF administration protocols. In this context, the question of the potential of CLE for non-contrast-enhancing tumors has been raised additionally. Non-contrast-enhancing tumors show an intact blood brain barrier and therefore no or very little extravasation of SF into the tumor nor the brain parenchyma, thus not suitable for CLE. With injection of SF after brain incision, CLE may pave the way to identify tumor margins in tumor entities showing intact BBB. Investigations thereof are currently ongoing.

Another issue raised by the community is a proper reading and understanding of the cytoarchitectural characteristics of the CLE images. Hereby, a better understanding of the underlying pharmacodynamics of SF in different types of tissue or tumor types would be beneficial and further comparison with conventional histological methods is needed. Work is ongoing in cross-correlating CLE images with classical H&E images. Parallels and differences are widely discussed and analyzed among the pathologists of the user community.

Validation of CLE against other modalities like magnetic resonance imaging-based navigation or diffusion tensor imaging fiber tracking, which all lose accuracy during surgery, or even the potential of CLE to enable re-calibration of the navigation, are subjects of further investigations raised by the community. Moreover, CLE for vessel formations, although addressed by some authors (27), needs further validation. Other aspects that the community raised are that the system could potentially contribute to an improved selection of specimens for cyto- or histological examination (“sampling quality control”) and that SF extravasation and uptake patterns could potentially improve understanding of the tumor environment *in vivo* or serve as a biomarker to support intraoperative diagnosis.

In conclusion, considering also the possible future applications that this machine may demonstrate in neurosurgery, the analyzed publications show promising diagnostic performance of CONVIVO compared to standard methods in histopathology. Nevertheless, the multitude of used SF protocols and the conditions investigated still warrant a better understanding of the method and its application in neuro-oncology and a further optimization of SF protocols for CLE, and we feel that this is one of the main points that future investigations may have as a main objective. At this time, three centers are running larger clinical trials (a multicenter trial in Germany: INVIVO, NCT04597801 (52); a trial in Berne, Switzerland: CLEBT, NCT04280952 (53); a trial in Milano, Italy: Besta Institute Review Board, verbal n. 72/2020), focusing on the concordance of CLE with definitive histopathological analysis. Their results will help to further improve SF protocols in the various tumor entities investigated. Non-inferiority when comparing CLE with current diagnostic standards, such as frozen section, is a further criterion required prior to positioning the method in routine clinical practice. Data from similar *in vivo* trials performed with the CONVIVO already show promising results (24) and more clinical data are expected from the three ongoing trials. Moreover, further data from studies analyzing “vascular” applications of CONVIVO are still lacking, as we feel that CONVIVO might have potentiality in assessing qualitatively and quantitatively blood flow in a specific vessel of interest, rendering the technology of high interest also during neurovascular procedures such as clipping of aneurysms, removal of arteriovenous malformations and performing bypasses. Looking at the oncological purposes, once appropriate protocols for the different use cases will be determined and the proof of accuracy provided, also with the possible help of Big Data technology (54), specific classification systems will need to be defined to ensure standardized diagnostic criteria and to establish a common language among the clinical users, favoring the system to enter routine clinical practice.

## Author contributions

Conceptualization, FR, AM, KQ and FA, methodology, FR and KQ, investigation, FR, AM, JH, EM, KQ, BP and FA, data curation, FR, KQ, EM, writing, original draft preparation, FR, AM, KQ writing, review and editing, FR, AM, KQ, EM, JH, BP, FA, supervision, FA All authors contributed to the article and approved the submitted version.

## Funding

This research is partially funded by Carl Zeiss Meditec AG, Oberkochen, Germany.This work was partially supported by the Associazione Paolo Zorzi per le Neuroscienze Onlus and by the Italian Ministry of Health (RRC).

## Conflict of interest

KQ receives fees for coordinating the community of researchers and clinicians working with the ZEISS CONVIVO device.

The remaining authors declare that the research was conducted in the absence of any commercial or financial relationships that could be construed as a potential conflict of interest.

## Publisher’s note

All claims expressed in this article are solely those of the authors and do not necessarily represent those of their affiliated organizations, or those of the publisher, the editors and the reviewers. Any product that may be evaluated in this article, or claim that may be made by its manufacturer, is not guaranteed or endorsed by the publisher.
