# Supplementary material for: Confocal laser imaging in neurosurgery: A comprehensive review of sodium fluorescein-based CONVIVO preclinical and clinical applications
Source: Front Oncol. 2022 Oct 3;12:998384. doi: 10.3389/fonc.2022.998384 (PMC9574261; doi:10.3389/fonc.2022.998384)
Supplement: Supplementary file 1 [file Table_1.docx]

| **study** | **study type** | **CLE system** | **SF protocol**  **imaging protocol** | **fluorophore re-administration** | **number of cases** | **imaged tumor entities** | **key study data** | **diagnostic performance** |
| --- | --- | --- | --- | --- | --- | --- | --- | --- |
|  | *human/animal*  *in-vivo/ ex-vivo* | *device name*  *manufacturer* | *dosage, administration route, timing of administration*  *timing of imaging after SF administration* | *dosage and timing of SF re-administration* | *no. of patients or animals*  *no. of biopsies* | *confirmed histological tumor types* | *objectives*  *main findings* | *sensitivity, specificity, diagn. accuracy, where applicable* |
| Acerbi et al., 2020(29) | human, prospective, ex-vivo | ZEISS CONVIVO | 5 mg/kg i.v., at anesthesia induction  CLE images recorded between 84-214 minutes after SF administration | not re-administered | 15 patients with 60 biopsies in total | 15 HGGs | study objectives:  to study the accuracy of the ZEISS CONVIVO CLE system in giving an intraoperative diagnosis  main findings:  First study to prospectively assess the ability of the CONVIVO device in obtaining intraoperative diagnosis and categorizing morphological patterns at both central core and tumor margins, based on a near real-time, blinded interpretation of the pathologist during surgery in the OR | diagnostic accuracy CLE vs. frozen sections:  obtaining a diagnosis   - 80% at central core - 80% at tumor border   categorizing morphological patterns:   - 93.3% at central core - 80% at tumor margins   accuracy CLE vs. permanent sections:  obtaining a diagnosis:   - 80% at central core - 67% at tumor border   categorizing morphological patterns:   - 87% at central core - 67% at tumor margins |
| Abramov et al., 2021(33) | human, retrospective, ex-vivo | ZEISS CONVIVO | 2 or 5 mg/kg i.v., at anesthesia induction  CLE images recorded at:   - single-dose group: 123.2 ± 35.9 minutes (range: 90 - 180) - initial-dose group: 93.9 ± 50.1 minutes (range: 13 - 180) - redose group: 6.4 ± 3.9 minutes (range: 2 - 15)   after fluorescein administration | 5 mg/kg i.v.  when CLE image brightness was considered inadequate by the neurosurgeon (in 6 cases)  CLE images recorded 6.4±3.8 minutes after SF following re-administration | 47 patients with 49 biopsies  re-administration group: 6 cases, 12 imaging biopsies | re-administration group:  4 gliomas  1 metastasis  1 choroid plexus carcinoma | study objectives:  to analyze the differences in dosing strategies, diagnostic interpretation, and fluorescence signal strength and decay in addition to image quality  main findings:  the brightest and most contrasting images were observed in the re-administration group compared to the initial-dose and single-dose groups. Different doses of SF did not significantly affect brightness. As the mean timing of imaging increased, the percentage of accurately diagnosed images decreased. | diagnostic accuracy:   - redose group:   - 83% irrespective of CLE experience - initial-dose group:   - 72% (CLE-experienced reviewers)   - 39% - 61% (CLE-inexperienced reviewers) - single-dose group:   - 47% (CLE-experienced reviewers)   - 53% - 63% (CLE-inexperienced reviewers)   mean ± scores for ​​qualitative assessment of images on a 1-to-5 scale:   - redose group: 4.5 ± 0.6 - initial-dose group: 2.3 ± 0.2 - single-dose group: 2.3 ± 0.1   as the mean timing of imaging increased, mean brightness and contrast of the images and the percentage of accurately diagnosed images decreased |
| Belykh et al., 2018(31) | human, retrospective, ex-vivo  and  mouse model, in vivo | ZEISS CONVIVO | animals:  2-5 mg/kg i.v. via tail vein 5 minutes prior to imaging  humans:  2-5 mg/kg iv 5-60 minutes before imaging | not re-administered | 31 human cases total, 22 cases with SF | 9 HGGs  4 metastases  3 Schwannomas  3 meningiomas  1 LGG  11 other tumors | study objectives:  to describe an extension of a CLE imaging modality that produces Z-stack images and three-dimensional (3D) pseudo-colored volumetric images | not applicable |
| Belykh et al., 2020(28) | human, prospective, ex-vivo | ZEISS CONVIVO | gliomas and meningiomas: 2 mg/kg i.v., at anesthesia induction  metastatic lesions: 5 mg/kg i.v., at anesthesia induction  in one case: 40 mg/kg, at induction of anesthesia | 5 mg/kg  for contrast improvement during surgery if deemed necessary by the neurosurgeon  re-administration occurred at a mean of 157±52 min. after the first administration | 47 | cases with a single dose:  29 HGGs (19 primary, 5 recurrent, 5 infiltrating gliomas)  3 LGGs  7 meningiomas  4 metastatic brain lesions  1 choroid plexus carcinoma  1 craniopharyngioma  1 schwannoma  1 arteriovenous malformation (reactive normal brain)  cases with redosing:  5 gliomas  1 metastasis  1 choroid plexus carcinoma | study objectives:  to assess the feasibility and diagnostic accuracy of CLE optical biopsies of brain lesions to identify relevant practical and methodologic variables for future in vivo use in clinical studies  main findings:  detailed quantitative and descriptive analysis of image features for different brain tumors; first time where a second SF injection was used to improve diagnostic power of CLE; blinded analysis of CLE images by neuropathologist and CLE-experienced / inexperienced neurosurgeons  reinjection of SF feasible: improved brightness / contrast and overall image quality. SF reinjection increased the percentage of images with an accurate diagnosis from 67% (18/27) to 93% (14/15), and decreased the percentage of nondiagnostic CLE cases from 26% (7/27) to 13% (2/15)  A decrease in image quality was found for biopsies when the SF was injected 1 - 5 minutes before imaging. | diagnostic accuracy across all cases by CLE experience and specialty:  CLE-experienced neuropathologist:   - 72% sensitivity - 90% specificity - 97% positive predictive value - 38% negative predictive value   CLE-experienced neurosurgeon:   - 74% sensitivity - 92% specificity - 97% positive predictive value - 47% negative predictive value   CLE-inexperienced neurosurgeon:   - 80% sensitivity - 60% specificity - 71% positive predictive value - 72% negative predictive value   a breakdown by tumor type contained in original publication |
| Belykh et al., 2018(26) | animal / mouse model, in-vivo and ex-vivo | ZEISS CONVIVO | 0.01 and 1 mg/ml i.v.  CLE images recorded 15-60 minutes after SF administration | not re-administered | 10 mice  90 biopsy sites | rodent glioma model  using GL261-Luc2 mouse glioma cells | study objectives: to investigate the diagnostic accuracy of in vivo CLE to differentiate normal brain, injured normal brain, and tumor tissue in an animal glioma model.  main findings:  successful differentiation between normal, injured, and tumor brain tissue in mouse glioma models | differentiation of tumor from non-tumor tissue:   - 90% mean accuracy - 86% sensitivity - 96% specificity - 98% positive predictive value - 79% negative predictive value   high interobserver agreement (Cohen’s kappa, k = 0.74)  The percentage of correctly identified images was significantly higher for images with a quality rating > 5 than for images with a quality rating ≤ 5 (scale 1 - 10).  Image quality did not differ in the subgroups of the 2 SF concentrations, median image quality scores:   - 7 for the 0.1 mg/ml group - 8 for the 1 mg/ml group   Although the 0.1 mg/ml concentration of SF produced adequate CLE images, the 1 mg/ml concentration resulted in brighter images overall, which provided more pronounced, clear, and consistent staining patterns. |
| Belykh et al., 2019(25) | animal / mouse model, in-vivo and ex-vivo | Optiscan 5.1 (Carl Zeiss AG, generation 1 device)  ZEISS CONVIVO (generation 2 device) | escalating doses of 0.1, 1, 2, 5, 8, 20, 40 mg/kg i.v.  CLE images recorded 5 - 120 min. after SF administration | not re-administered | 37 | rodent glioma model  using GL261-Luc2 tumor cells | study objectives:  investigation of performance improvements of generation 2 compared to generation 1 CLE devices; investigation of optimal imaging settings for the generation 2 device.  main findings:  compared generation 1 and 2 CLE devices in identifying normal brain tissue, vasculature and tumor cells; assessed imaging parameters (gain, laser power, brightness, scanning speed, imaging depth, and Z-stack/3D image acquisition) and evaluated optimal values for better neurosurgical imaging performance with the generation 2 device. | Different concentrations of intravenous The generation 2 device had a smaller field of view, but higher image resolution, and sharper, clearer images.  CLE imaging with SF allowed identification of nuclear and cytoplasmic contours in tumor cells.  Bolus injections of higher concentrations of SF resulted in brighter images of tumors with less noise, and an overall increase in the fraction of diagnostic frames, compared to lower SF concentrations.  20 and 40 mg/kg vs. 0.1–8 mg/kg resulted in better image clarity and structural identification. The best working dosages were 20 and 40 mg/kg. |
| Belykh et al., 2021(27) | animal / swine model, in vivo and ex vivo  human, retrospective), in-vivo and ex-vivo | animals:  ZEISS CONVIVO  (generation 2 device)  humans:  OptiScan 5.1  (generation 1 device, equivalent to the generation 2 device) | animals:   - 1, 2, 5 mg/kg i.v. - 0.005%, 0.05%, 0.1% in 5 ml intracarotid - to visualize meningeal lymphatic vessels: 4.2 mg (0.42 ml of a 10 mg/ml solution), in subarachnoid space   humans:  500 mg i.v. (5 ml of a 10% solution), 5 minutes prior to CLE imaging | redosing is mentioned, no further details provided in article | 20 human cases | human cases:  3 astrocytomas WHO II  4 oligodendrogliomas WHO II  3 invasive astrocytomas WHO III  7 oligoastrocytomas WHO III  3 glioblastomas WHO IV | study objectives:  qualitative and quantitative analysis of blood flow in normal and pathologic brain and spinal cord microvasculature; to assess the feasibility of using a CLE system to visualize blood cells inside vessels and perform continuous blood flow imaging after the fluorescent contrast injection  main findings:  Brain and spinal cord microvasculature and blood flow were visualized in all experimental animals. Erythrocyte flow observed in vessels 5-500 μm in diameter; thrombosis, flow arrest and redistribution, flow velocity changes, agglutination, and cells rolling could be assessed in normal and injured brain tissue. CLE allowed visualization of intravascular blood flow significantly longer than the widefield operating microscope. Lymphatic vessels were also visualized. | Animals:  Contrast created by fluorescein in the intravascular compartment, in the vessel wall, and in the perivascular parenchyma allowed appreciation of the vessel wall cellularity. Arteries could be differentiated from veins. The functional status of precapillary vessels could be observed. The spinal cord microvasculature was also easily visualized.  Intravascular imaging was possible for up to 30 minutes after a 1 mg/kg intravenous dose of fluorescein, and for more than 3 hours following subsequent fluorescein injections.  Visualization of the vessel wall was possible earliest 8 minutes after fluorescein injection, and was more common at approximately 30 minutes after injection. Tissue injury, contrast extravasation, and additional injections of fluorescein all made visualization of the vessel wall easier and made the vessel wall more obvious at later imaging times.  Humans:  CLE imaging demonstrated abnormal tissue architecture with profound extravasation of the contrast medium.  Microvasculature could be differentiated as normal and abnormal in both in vivo and ex vivo images.  Dural lymphatic channels could be discriminated against blood vessels and revealed dural cellular architecture and organization. |
| Höhne et al., 2021(36) | human, retrospective, in-vivo | ZEISS CONVIVO | 5 mg/kg i.v., intraoperatively 10-120 minutes before imaging | not re-administered | 12 cases | 5 metastases (1 lung cancer, 2 melanoma, 1 unspecified carcinoma, 1 colon cancer)  4 GBM WHO IV  1 oligodendroglioma WHO II  1 neurocytoma WHO II  1 gliosis/recurrent hemangiopericytoma | study objectives:  to evaluate the feasibility, safety, and potential applications of CLE imaging, to provide intraoperatively acquired CLE images with matched frozen section analysis, microscope photographs, and intraoperative navigation  main findings:  CLE was found to be beneficial in terms of high-quality visualization of fine structures and for displaying hidden anatomical details. Yellow-green fluorescent macroscopic staining of the tumor tissue was observed in all patients and was considered helpful guidance in all cases. A shorter elapsed time between SF administration and imaging resulted in more assessable images. | CLE was integrated seamlessly into the surgical flow. It could be safely performed, as the small and versatile probe mirrors any other microsurgical instrument that is held in one hand and inserted into the cavity without traumatizing healthy tissue.  A shorter elapsed time between sodium fluorescence administration and imaging led to more asses. sable images. Regarding image quality, no major differences with respect to the timing of sodium fluorescence injection were noted. Images from 11/12 cases were recorded within 90 minutes of SF administration, which has been shown to be an optimal imaging window in other publications.  SF fluorescence was detected using CLE in:   - 100% of cases at the tumor border - 92% of cases in the tumor center - 58% of cases in the perilesional zone   SF and CLE imaging using the ZEISS CONVIVO device were considered safe and feasible. |
| Belykh et al., 2020(34) | human, prospective, ex-vivo | ZEISS CONVIVO | 2 mg/kg i.v., 1 - 45 minutes before imaging | not re-administered | 9 cases | 9 pituitary adenomas  with a total of 19 digital CLE biopsies | study objectives: to examine the feasibility of CLE with SF for rapid intraoperative assessment of human pituitary adenoma tissue microstructure  main findings:  The configuration of the imaging probe allowed access through the transnasal transsphenoidal corridor in cadaveric specimens. CLE could be used to visualize histopathologic characteristics of pituitary adenomas. The optimal timing for SF administration at 2 mg/kg was between 1 - 10 minutes prior to imaging, too early or late administration resulting in suboptimal contrast and consequently nondiagnostic images. | On a per case basis, successful tumor identification was possible in:   - 100% (7/7) of cases with frozen sections - 88% (7/8) of cases with permanent histology - 100% (9/9) cases with CLE imaging   study did not assess diagnostic accuracy, but reported a qualitative assessment of CLE images relative to frozen sections and permanent histology  overall description of tissue in CLE:   - including nondiagnostic images: 57.9% (11/19) of CLE biopsies were described as pituitary adenoma, 42.1% (8/19) as nondiagnostic - omitting nondiagnostic images: 100% (11/11) of CLE biopsies were described as pituitary adenoma   The reasons for nondiagnostic CLE images were biopsy acquisition < 1 minutes or > 10 minutes after fluorescein injection (n = 5) and blood artifacts (n = 1). In addition, very small biopsy specimens made it difficult to find optimal imaging locations.  assessment of CLE images relative to frozen sections:  from the total of 16 biopsies classified as “definitive” (13) or “favoring” (3) for pituitary adenoma, 62.5% (10/16) were concordant in CLE   - of 13 biopsies with definitive pituitary adenoma in frozen section: 61.5% (8/13) concordant CLE biopsies, 38.5% (5/13) nondiagnostic CLE biopsies - of 3 biopsies favoring pituitary adenoma in frozen section: 667% (2/3) concordant with CLE, 33.3% (1/3) nondiagnostic   assessment of CLE images relative to permanent histology:  from a total of 12 biopsies classified as “definitive” (7) or “favoring” (6) for pituitary adenoma, 7 were concordant in CLE (53.8%)   - of 7 samples with definitive pituitary adenoma in permanent histology: 4 concordant CLE biopsies (57.1%), 3 nondiagnostic CLE biopsies (42.9%) - of 5 samples favoring pituitary adenoma in permanent histology: 3 concordant CLE biopsies (60%), 2 nondiagnostic CLE biopsies (40%)   Of 4 nondiagnostic samples in permanent histology: 3 concordant CLE biopsies (75%), 1 pituitary adenoma CLE biopsy (25%). |
| Belykh et al., 2021(35) | human, case report from prospective study, ex-vivo | ZEISS CONVIVO | 40 mg/kg i.v., shortly after induction of anesthesia  CLE images recorded up to 90 minutes after SF administration | not re-administered | 1 case | 1 anaplastic oligodendroglioma, non-enhancing in MRI, IDH-mutant, 1p/19q co-deleted, with ATRX expression in vast majority of lower grade areas, WHO grade III (discrete foci of hypercellularity and increased mitotic figures despite large low grade regions) | study objectives: case report to document the concurrent use of wide-field fluorescence-guided surgery and CLE with high-dose SF for intraoperative visualization of tumor tissue cellularity in a nonenhancing glioma  main findings: the higher dose of 40 mg/kg SF produced unusually excellent contrast and revealed fine intraoperative imaging characterization for areas of hypercellularity and tissue | CLE with high-dose SF provided extremely clear images of cellular architecture, mitotic figures, endothelium of vessels, and swollen axons. The brightness and clarity of the CLE images revealed a distinct morphologic appearance not typically observed with lower-dose SF, especially 90 minutes after administration. The patient tolerated this dose well, and the patient’s postoperative yellowish skin discoloration resolved rapidly.  The relatively high dose of SF may produce a significant benefit in identifying tumors not labeled well with lower doses during fluorescence-guided surgery and CLE imaging. |
| Schebesch et al., 2019(32) | human, case reports, ex vivo | ZEISS CONVIVO | 5 mg/kg i.v., administration time not indicated | not re-administered | 3 cases | 1 supratentorial astrocytoma WHO III, 1 motor area glioblastoma WHO IV, 1 oligodendroglioma WHO grade III | study objectives: to present the authors’ institutional algorithm of combining different neuroimaging modalities for surgical neuro-oncological procedures (including CLE)  main findings: … the implementation of the institutional algorithm has not only greatly contributed to the significantly prolonged PFS and OS, but has also clearly increased the rate of gross-total resections of HGG | SF impressively visualized the tumor … ex vivo. |
| Abramov et al., 2022(24) | human, prospective, in-vivo | ZEISS CONVIVO | 5 mg/kg i.v., ≤ 5 minutes before imaging at surgeon’s request  CLE images recorded between 5 - 193 minutes after SF administration | not re-administered | 30 cases  31 tumors: 26 contrast enhancing in preoperative MRI, 5 non-enhancing | 7 GBM WHO IV (5 recurrent, 2 primary), 3 anaplastic astrocytoma WHO III, 1 anaplastic oligodendroglioma WHO III, 1 pilocytic astrocytoma WHO I, 1 subependymoma WHO I, 1 meningothelial meningioma WHO I, 1 fibrous meningioma WHO I, 1 pineocytoma, 1 hemangioblastoma, 1 acoustic schwannoma, 1 choroid plexus papilloma, 1 perineurioma, 1 mature teratoma, metastases: 1 breast adenocarcinoma, 1 renal cell carcinoma, 1 lung adenocarcinoma, 4 cases with reactive brain tissue | study objectives: to evaluate the safety and feasibility of using the first clinical-grade CLE system using fluorescein sodium for intraoperative in vivo imaging of brain tumors  main findings: Overall, 10,713 CLE images from 335 regions of interest were acquired, of which 46% were interpretable. interpretable CLE images were obtained from all patients. The first interpretable image was obtained within a mean of 6 images. The mean time to interpretable image acquisition was 5 seconds after CLE initiation. Mean duration of CLE use per case was 7 minutes. The number of interpretable images per patient improved as the study progressed. The percentage of interpretable images correlated with the cumulative duration of CLE use and the duration of CLE use per case. Deeply located regions of interest were associated with a higher percentage of interpretable images compared with superficial regions. The surgical telepathology software platform used in 11 cases was rated highly for communication, guidance, efficiency, and coordination with the neuropathologist. | CLE vs. frozen section (all tumors):   - accuracy: 94% - sensitivity: 94% - specificity: 100% - PPV: 100% - NPV: 67%   CLE vs. permanent histology (all tumors):   - accuracy: 92% - sensitivity: 90% - specificity: 94% - PPV: 97% - NPV: 81%   CLE vs. permanent histology (glioma):   - accuracy: 93% - sensitivity: 91% - specificity: 100% - PPV: 100% - NPV: 78%   CLE vs. permanent histology (reactive brain):   - accuracy: 92% - sensitivity: 0% - specificity: 90% - PPV: 0% - NPV: 90%   Histology images could be matched with the CLE images in 97% (29/30) of cases. Histology and CLE images of perineurioma could not be well matched. |
